# Strategies to build more effective interventions for elder abuse: a focus group study of nursing and social work professionals in Hong Kong

**DOI:** 10.1186/s12877-022-03682-4

**Published:** 2022-12-19

**Authors:** Elsie Yan, Louis To, Debby Wan, Xiaojing Xie, Frances Wong, David Shum

**Affiliations:** 1 Department of Applied Social Sciences, The Hong Kong Polytechnic University, Hong Kong SAR, China; 2 School of Nursing, The Hong Kong Polytechnic University, Hong Kong SAR, China; 3 Faculty of Health and Social Sciences, The Hong Kong Polytechnic University, Hong Kong SAR, China

**Keywords:** Elder abuse, Intervention, Focus group, Practice

## Abstract

**Background:**

One in six older adults living in communities experience abuse and neglect. Elder abuse has serious consequences for individuals, families, and society, including mortality, physical and psychological morbidities, and increased care requirements. Timely and effective interventions for elder abuse should therefore be a priority. This study used a qualitative focus group approach to address the following questions: What are the essential elements of elder abuse interventions? What can be done to improve current interventions?

**Method:**

The 32 participants in this focus group study included social workers, medical social workers, and nurses from seven organizations who shared their knowledge and insights. All sessions were conducted online, audio-recorded, and transcribed verbatim. Three researchers with backgrounds in social work and psychology independently coded the transcripts and agreed on the themes emerging from the focus groups.

**Results:**

Based on the experiences of frontline helping professionals in Hong Kong, we highlighted the key factors for effective elder abuse intervention: 1) identification and assessment; 2) essential skills and attitudes; 3) elements of effective interventions; 4) collaborative efforts across disciplines and agencies; and 5) raising awareness among professionals and the public.

**Conclusions:**

Training can equip frontline professionals with the necessary skills to identify elder abuse cases and to assess the risk of abuse. Effective interventions should not only address clients’ safety and need for tangible support but also respect their autonomy and privacy. A client-centered, strength-based approach that involves supportive peers and addresses the complex family relationships involved can be useful. Interventions should also involve cross-discipline and cross-agency collaboration.

## Background

Elder abuse is “a single, or repeated act, or lack of appropriate action, occurring in any relationship where there is an expectation of trust, which causes harm or distress to an older person” [1]. It can occur in any socioeconomic and ethnic group. A review of 52 studies from 28 countries found an average prevalence rate of 15.7% [2]. Abuse has serious consequences for individuals, families, and society, including mortality, physical and psychological morbidities, and an increased need for care [3].

Due to its prevalence and detrimental consequences, efforts have been made to develop intervention strategies for elder abuse. These include community support for vulnerable older adults [4] and caregivers [5,6], telephone helplines [7,8], emergency shelters [9], and multidisciplinary case management [10,11,12].

### Problems with current prevention and intervention efforts

Recruiting and retaining participants in elder abuse interventions has proven difficult [13], which may be due to various factors. Victims of abuse who depend on their abusers for care and companionship may accept mistreatment through “tactic exchange” [14,15]. The abusers may have larger social networks than their victims, who thus perceive them to have more power, which can reduce the likelihood of victims seeking assistance [14,15]. Older adults may also be unaware of the available community resources. Population-based research in the U.S. suggests that only between 4% and 14% of elder abuse victims are aware of the agencies through which they can make formal responses, such as adult protective services and law enforcement [14,15,16]. In addition to the lack of knowledge about available resources [17], traditional cultural values, the wish to protect family honor and maintain harmony, and a general lack of trust in third-party intervention represent barriers to elder abuse victims seeking assistance [17].

The perspectives of victims [18] and of professionals have been considered in the literature in terms of types of intervention [19] and the reporting of suspected elder abuse [20]. We aim to extend these studies by drawing on the experience of frontline professionals to identify the essential elements in a range of elder abuse intervention strategies.

### The Hong Kong context

In 2004, the Hong Kong government first initiated a study on elder abuse, and the Social Welfare Department published guidelines for tackling elder abuse and established an online platform for reporting instances of abuse. Very few cases were reported between 2005 and 2022, ranging from 319 to 627 annually, with most being instances of physical abuse. However, a prevalence rate of 27.5% has been reported among community dwelling older adults [21], so the reported cases only represent the tip of the iceberg.

In terms of legislation, reporting elder abuse is not mandatory in Hong Kong, either for lay persons or professionals. Amendments to the Domestic Violence Ordinance (Cap. 189), originally aimed at protecting women and children in a marital relationship, were made in 2008. This was renamed the Domestic and Cohabitated Relationships Violence Ordinance (Cap. 189), and now is aimed at protecting all individuals living in the same household, including elderly victims.

The responsibilities of taking care of older people fall to a great extent on their families. The traditional cultural values of “filial piety” prescribe that adult offspring take care of their aged parents. The government also takes an “aging in place” approach, and encourages family care. Those providing care to a senior family member can apply for a tax reduction of around US$800, and Hong Kong citizens co-residing with a family member age 60 or above have priority when applying for public housing. In addition, only 8.5% of those aged 65 or above in Hong Kong live in long-term care facilities, as availability is limited [22]. Thus, some households in Hong Kong hire domestic help to assist with elder care. In 2019, 400,000 foreign domestic helpers worked in Hong Kong. Many receive a wage of around US$600 per month and are required by law to live in their employers’ residence. They perform caregiving tasks such as cooking, cleaning, transferring, bathing, and feeding. In addition to this heavy workload, many experience language barrier difficulties when communicating with care recipients.

This study attempted to identify essential elements of elder abuse interventions from a series of focus groups with social and health care professionals in Hong Kong.

## Methods

### Participants

We recruited a sample of 32 participants from seven local hospitals and governmental and nongovernmental organizations. They included social workers who focus on senior support (*n* = 7), domestic violence intervention (*n* = 4), shelters for elder abuse victims (*n* = 9), medical support (*n* = 3), and nurses in accident and emergency hospital units (*n* = 9). The participants were first recruited through the research team’s professional networks. Community collaborators were invited to refer frontline colleagues with no less than five years’ experience in elder care, domestic violence, and elder abuse to our study. We took a snowballing approach to recruitment, and participants from the first round of focus groups were invited to refer colleagues concerned about elder abuse issues, again only if they had five years or more of experience. Participants’ work settings and backgrounds are summarized in Table 1.

**Table 1:** Participants’ Backgrounds and Work Settings

	Professionals	Number of Participants (N = 32)
Community centers for seniors	Social worker	7
Shelter for elder abuse victims	Social worker	9
Domestic violence intervention agencies	Social worker	4
Hospital medical social work units	Medical social worker	3
Hospital accident and emergency units	Nurse	9

The research protocol was approved by the research ethics committee of the authors’ university. All participants provided verbal consent to participate before the focus group commenced.

### Procedures

Our qualitative approach of using focus groups had to be conducted online due to the social distancing requirements of the COVID-19 pandemic. The participants shared their experience and knowledge of elder abuse intervention through one of the eight group sessions, each of which was 120 to 180 minutes long with between 3 and 7 participants in each. Researchers in this study served the role of focus group facilitators and encouraged participants to share their views in the groups. Researchers did not actively shared their own views to avoid bias.

The sessions were all audio recorded and transcribed verbatim. All transcripts were reviewed by the team to ensure accuracy. Microsoft Excel was used for assigning codes and statements were compared across and within groups [23]. Three researchers read the transcripts and independently created preliminary code lists. Codes representing similar meanings were grouped into broader thematic categories to create a coding scheme. Any differences in the three researchers’ coding were resolved by discussing the context and meaning of the statement and agreeing on a final code. The authors then conducted a final review of the themes and subthemes.

We invited ten of the participants to comment on the themes generated from the focus groups, and they agreed that the themes accurately reflected their opinions.

Appendix I presents the focus group guides used in the present study.

## Results

The participants highlighted service gaps and areas requiring improvement in their discussions of elder abuse interventions. In the next section, we address the following key themes that emerged from the discussions: 1) identification and assessment; 2) essential skills and attitudes; 3) effective interventions; 4) collaborative efforts; and 5) raising elder abuse awareness. Table 2 summarizes the themes and subthemes generated from the focus groups. To protect participants’ identity, pseudonyms are used throughout this paper.

**Table 2:** Key Themes and Subthemes

**Key Themes**	**Subthemes**
1. Identification and assessment	Avenues for case identification Worker awareness and readiness Organizational culture and support Risk assessment Comprehensive case assessment
2. Essential skills and attitudes	Respecting client autonomy and privacy Rapport building Active listening Progressing at the client’s pace
3. Effective interventions	Providing tangible support Strength-based approach and post-traumatic growth Support and self-help groups Addressing complicated family relations Supporting caregivers Follow-up support
4. Collaborative efforts	Multi-disciplinary and cross-agency collaboration Effective use of community resources
5. Raising elder abuse awareness	Professional training Public education Addressing the bystander effect

## Identification and assessment

Timely and effective case identification is the first step in any elder abuse intervention. The participants suggested effective detection and identification approaches.

## Avenues for case identification

Elder abuse victims may not proactively seek help, and thus professionals should inquire about issues relating to abuse when on routine service visits.

We always ask for additional information when [clients] apply for other services [unrelated to elder abuse]. We only learn about an abusive situation when they tell us more. Clients very rarely disclose that they are being abused when they first come to us. (Amelia, Social Worker, Elderly Services)

Many people do not come in seeking elder abuse services. They may need someone to escort them to medical appointments, need help with household chores, have financial difficulties, difficult relationships with family members, etc. It is only when we dig deeper that we realize it is an elder abuse case. Very few people will tell you spontaneously that someone in their family hit them. (Carmen, Social Worker, Elder Abuse Shelter)

Participants also noted that involving “knowledgeable others” in elder abuse detection is important. They suggested that those in close contact with older people, such as neighbors, janitors, security guards, and district councilors, can help in identifying cases of elder abuse. Most Hong Kong residents live in apartments, and hundreds of households are typically packed into the same building. This unique living environment means that neighbors and building security guards are in a very good position to detect and identify elder abuse cases, as they have frequent day-to-day contact with older residents.

Security guards in residential buildings have lots of opportunities to communicate with older residents. Workers on food delivery teams only contact them if they are using their services, and many would avoid mentioning suspected elder abuse for fear of getting themselves into trouble. If we could provide some training to security guards, they could easily keep an eye on older residents while they are on duty. (Amelia, Social Worker, Elderly Services)

The participants also observed that medical contexts offer an effective avenue for elder abuse identification. Older people often regularly visit their family doctors or hospitals. Frontline medical professionals must therefore be aware of and sensitive to elder abuse issues:

I once saw an old woman in the triage station with both hands scalded by hot water. It was pretty abnormal that it was both hands, so I suspected that someone had done it to her. When asked about the injuries, she eventually told me that her son had scalded her. We do that with unusual injuries, either nurses at the triage station or physicians in the examination rooms. (Iris, Nurse, Hospital Accident and Emergency Unit)

### Awareness and readiness

Following this, the participants also noted that the readiness to address potential cases of elder abuse is critical for its detection.

A client may have many internal struggles; they may feel too ashamed to tell us what happened. Under such circumstances, social workers’ readiness is of critical importance. If a worker chooses to turn their back, the client will just continue to hide the abuse. If the worker is willing to listen, however, and is ready to dig deeper into the issue, clients will be more willing to share and positive change can happen. Social workers need to know themselves, to be ready all the time, and to continually reflect on the reasons why they might avoid addressing elder abuse issues. (Carmen, Social Worker, Elder Abuse Shelter)

### Organizational culture and support

Participants suggested that organizational culture and policy may also influence the willingness and motivation to identify elder abuse:

When the attending doctor sees that an injury is in an uncommon location, he or she will always consult a more senior doctor who specializes in the study of suspected elder abuse. The Hospital Authority has specific guidelines requiring that every hospital has at least one medical doctor or nurse responsible for handling suspected elder abuse cases. We work with them very closely to determine whether the case is elder abuse or just a regular accidental injury. (Liz, Medical Social Worker, General Hospital)

### Risk assessment

The primary goal of elder abuse intervention is to ensure client safety. The participants discussed the importance of risk assessment:

From the moment an older client contacts us, regardless of whether he or she comes in person or contacts us by phone, we always start with a thorough risk assessment to ensure his or her safety, and are alert to any potential danger. (Helen, Social Worker, Shelter for Domestic Violence Victims)

We assess the case severity, whether a hospital visit is required, whether there have been similar incidents in the past, etc. This indicates how risky the current situation is. Especially in cases of long-term domestic violence, it could be extremely dangerous for us to leave the client at home with the perpetrator. (Gloria, Social Worker, Shelter for Domestic Violence Victims)

Those we interviewed rely on a range of methods, including quantitative assessment tools, examining case histories and clinical judgment, and assessing the risk level in individual cases. A comprehensive assessment requires a combination of objective and subjective measures.

### Comprehensive case assessment

After determining a client’s immediate risk and ensuring his or her safety, a comprehensive case assessment must be conducted:

All cases who use our shelter service complete the IMPACT scale assessment. We try to investigate how the abuse incident may have impacted the client: for example, sleep problems or their physical condition. We also provide older adults with Activities of Daily Living and Montreal Cognitive assessments to assess their physical and cognitive functions and basic self-care abilities. (Ofelia, Social Worker, Shelter for Elder Abuse Victims)

### Essential skills and attitudes

#### Respecting client autonomy and privacy

The participants acknowledge that elder abuse differs from other forms of domestic violence and that extra care is required in terms of clients’ autonomy and privacy. Studies focusing on Hong Kong have indicated that elder abuse victims prioritize family relationships, personal needs, and cultural considerations over their own physical and psychological safety [24]. This is supported by other studies suggesting that professionals should respect the autonomy of victims after giving them advice, even if their decisions are likely to expose them to unsafe situations [25].

Even if the client understands that he/she is being abused, we must still respect his or her wish as to whether any follow-up is needed, or whether we can bring legal action against the family member. (Wesley, Social Worker, Elderly Services)

Older persons really resent others knowing about their family problems. We take extra care to protect our clients’ privacy at our meetings. It is comforting for them to know that no one else will hear anything about our conversation. Without such reassurances, many would just leave. (Patty, Social Worker, Elder Abuse Shelter)

### Rapport building

The importance of building trust and a rapport with clients was also highlighted. Engaging elder abuse victims is essential for effective interventions, and studies in the U.S. have suggested that a relationship of trust can be developed through the engagement process and that strong client–practitioner relationships are likely to lead to desirable intervention outcomes [25].

Some clients may maintain that “it’s better to keep the skeleton in the cupboard,” but then after several meetings they decide that you are trustworthy and are willing to listen, and then they start talking. (Scarlet, social worker, elderly services)

Some clients may have a long history of “building a wall between themselves and the rest of the world” and are wary of other people. We need extra time and effort to build a relationship of trust with them before moving on. I feel that everything starts with trust: without trust, clients will only tell you superficial things … one small step at a time, it takes time. (Scarlet, Social Worker, Elderly Services)

### Active Listening

Active listening is the most commonly used therapeutic technique [26] in this area, and the participants identified active listening and patience as the two essential elements to support victims of abuse:

To build a rapport with a client, we need to put aside our presumptions and try to understand the person sitting in front of us as a unique individual with unique experience. (Patty, Social Worker, Elder Abuse Shelter)

It takes lots of courage to leave an abusive situation. Social workers should let clients know we are there to accompany them at every step. This would give them peace of mind about later changes. When a client finally opens up to share his or her stories, a story inevitably stirs up lots of tears, deep-seated feelings, and emotions. We need to provide clients with a safe space to tell their stories, both physically and psychologically. (Carmen, Social Worker, Elder Abuse Shelter)

### Progressing at the client’s pace

Male abuse victims in particular may resist services if they initially perceive a “helping” attitude:

We assume the role of friends or neighbors, merely there to show care, and slowly build up a friendly relationship. If we start off showing that we are there to help, that social workers are there to help you, it can hurt their ego and lead them to turn away. This is especially true for older males: some of them have quite big egos. (Scarlet, Social Worker, Elderly Services)

Workers may be inclined to bombard cases with information, telling them what sort of assistance and services are available, rushing into clinical assessment, etc. and that can be scary for clients. From my own experience, the “hard sell” approach can be a real turn-off. (Ofelia, Social Worker, Elder Abuse Shelter)

### Effective interventions

#### Providing tangible support

The participants said that many elder abuse victims rapidly leave the abusive situation with little preparation. They may require tangible support such as financial assistance, clothes, and shelter. Shelter services can provide support to victims in the form of day-to-day assistance or community services, in addition to a physically and psychologically safe environment [26,7]

Tangible needs are of the utmost importance to clients. Many residents in our shelter suffer financial hardships. They may have left home abruptly. Some only have their personal identity card with them, no mobile phone, no money, no clothes…Tangible support, like food from food banks, a television to keep updated on the news, warm clothes and bedding, etc., increases their sense of security. Their acceptance of such tangible support is a huge step for them, helping them to accept the changing situation. (Carmen, Social Worker, Elder Abuse Shelter)

Many elder abuse victims may not have a clear plan as to where they will go after they leave [their home]. Those who are with us [at the shelter] may not have all the necessary resources, so it is imperative that we provide basic necessities to them. Tangible support shows that we care about them. (Carmen, Social Worker, Elder Abuse Shelter)

### Strength-based approach and post-traumatic growth

A strength-based approach can also be taken, as suggested in various studies. Identifying victims’ strengths can help empower them and rebuild their identities, thus speeding up recovery [27,28], and the process of recovery may have a therapeutic effect and foster growth [29]. Our participants also observed post-traumatic growth in clients they have worked with:

Many clients who have left our shelters come back and visit every now and then. Apart from using our services, they actually want to contribute to society, to demonstrate their abilities. They volunteer to support the newcomers at the shelters and use their own experience to help other abuse victims. (Gloria, Social Worker, Shelter for Domestic Violence Victims)

To quote a client, “being abused at 60 years old is most unfortunate, but it’s just a phase.” There is still a long way ahead and he could still contribute to his family and society over the remainder of his life. (Florence, Center Director, Shelter for Domestic Violence Victims)

Many members of the Buddy Program1 share that they no longer feel ashamed about speaking of their experiences of abuse, and that they have transformed the feeling of shame into motivation to help other seniors who suffer abuse. (Patty, Social Worker, Elder Abuse Shelter)

### Support and self-help groups

Those working in shelter services reported making use of peer support programs to help elder abuse victims cope and heal.

We have an ambassador program for recruiting domestic violence survivors who have successfully overcome their trauma. Our clients not only struggle to help themselves; once they have moved on, they can jump out of their shadow and support others in difficulty. (Florence, Center Director, Shelter for Domestic Violence Victims)

Every now and then a client will say, “Just because other people can succeed doesn’t mean I can,” and continue to discount his or her own ability. But then it’s always nice to be able to refer to successful cases. Our clients very seldom share their abuse history in community centers, but they open up to fellow residents in the shelter, knowing that everyone here more or less has the same experience. It gives them hope. (Carmen, Social Worker, Elder Abuse Shelter)

### Addressing complicated family relations

The participants agreed that family dynamics in elder abuse cases are often complex, particularly when the abuser is a family member, and must thus be addressed in interventions. Older victims may value family relationships more than their own safety [25,17], particularly if they depend on the perpetrator in their daily lives [14]. They may also refuse to seek help from social services due to the traditional values that involve protecting the reputation of their family and thus their abuser [24].

They may have fought all the time; some [abusive2family members] may ignore the older person altogether. The older person is forced to roam the streets during the day and only return home late in the evening, just to avoid conflicts at home. This is psychological abuse if you ask me. Older persons react differently to situations like this. Some may avoid conflicts but still want to look out for the abusive family member. I have a case where the adult son is not taking care of the client in any way. The client goes out during the day to avoid his son but returns home to prepare meals for him day after day. The client didn’t want to leave and only came to us when it became unbearable. (Vicky, Center in Charge, Elderly Services)

Involving non-abusive family members in elder abuse interventions and providing education can help them recognize any issues. Other studies also demonstrate that supporting non-abusive family members can influence victims’ help-seeking behavior [14]

The abuser may not be easy to approach, so we can only start from other family members. (Denise, Social Worker, Elderly Services)

Families need to be educated about elder abuse, especially family members who are willing to offer help. We need to teach them about the serious impact of elder abuse and properly equip them with ways to handle it should it occur again. (Denise, Social Worker, Elderly Services)

### Supporting caregivers

The participants noted that caregiver interventions can help prevent elder abuse as caregivers often suffer from stress, particularly when care services are inadequate.

I have met carers who are under great pressure, and many may be incapable of providing adequate care, especially in dual-elder families. With the long waiting time for services, many caregivers feel they are trapped in a lion’s cage. This is a common and serious problem. (Beatrice, Social Work, Elderly Services)

We do not provide round-the-clock services. Even if we provide home help, it is for one or two hours at most, whereas family members are there 24 hours a day. It is worse if the care recipient is suffering cognitive decline. Some sleep very little and wander around at night making lots of noise, and their family still need to go to work the next morning. It can be very stressful to be trapped in this vicious cycle … and family members apply for elder care services because it becomes urgent. How is it helping if the waiting time ranges from one to three years? (Amelia, Social Worker, Elderly Services)

As mentioned, some households in Hong Kong hire foreign domestic helpers, who can also assist in preventing elder abuse. The participants in this study suggested that adequate training and support should be provided to these helpers:

There are quite a few older couples with a live-in domestic helper in our district. In these families, the main caregiver is the domestic helper. We do observe cases of borderline neglect every now and then. Available training and education programs mainly target family caregivers, but in many households the caregiving tasks are carried out by foreign domestic helpers. It is especially important to help those who are new to Hong Kong and new to caregiving tasks to establish a good relationship with their care recipients. A good start will help avoid many unnecessary misunderstandings. (Ursula, Social Worker, Elderly Services)

In addition to the formal programs, we also introduce foreign domestic helpers who have recently come to Hong Kong to our members who have some experience working here. This helps foster informal social support. There was once a case where an older care recipient fell and hit his head. The helper was very worried but was too afraid to tell her employer for fear that he would terminate her contract. We asked a fellow helper to talk to her and managed to find a solution that was agreeable to everyone. (Beatrice, Social Work, Elderly Services)

## Follow-up support

Elder abuse intervention should not end when the victim is discharged from hospital or leaves a shelter. The ultimate aim of such interventions is to enable the victim to lead a safe, meaningful, and sustainable life [24]. Some victims seek to maintain a relationship with their abuser [14]. The participants in our study outlined the follow-up support they provide to victims of elder abuse to ensure their safety, support them in their daily lives and, if required, help them to restore their relationships with their abusers:

Whether the older adult returns to his/her home or moves to a new community, we devise a safety plan. The plan covers their relationship with their abuser [and] managing emotional health, and includes the means to contact us at any time. They are fully informed that they can reach out to us any time should they have a problem or if they just want to have a casual chat with us. They may even choose to come back to the shelter. We follow up each case for a minimum of three months regardless of whether they are staying on their own, living in a nursing home, or have gone back to stay with their abuser. (Carmen, Social Worker, Elder Abuse Shelter)

We have a group therapy program catering for older adults who have left shelters. Most come to the shelter confused, there being simply too many things to manage, and they can spare very little time and energy for counseling, and some may not be psychologically ready for treatments. It is after we have settled all the practical issues such as housing and daily necessities that older adults have time to re-examine the abuse experience. (Carmen, Social Worker, Elder Abuse Shelter)

### Collaborative efforts

#### Multidisciplinary and cross-agency collaboration

The participants also highlighted the benefits of working with colleagues from other teams or disciplines. Such multidisciplinary approaches can enhance the efficiency of service delivery and improve intervention outcomes [25,7]. Our frontline practitioners agreed that no single profession can handle elder abuse cases alone and that input from a multidisciplinary team is helpful when delivering interventions.

Although there are social workers at the shelter to work on the case, we still see whether there is a family social worker from the community to follow up and make referrals if not. Social workers at shelters are responsible for client adjustment, daily necessities, [and] emotional and physical support. But when it comes to relationship and emotional problems with family members, we refer cases to social workers from the community for family counseling. (Rose, Social Worker, Elder Abuse Shelter)

In addition to those in social services, workers in healthcare, legal, and even financial services may encounter elder abuse victims [30]. Close collaboration and communication among such disciplines are thus essential. The participants in this study have experience of collaboration, but the process may not be smooth as points of view can differ. Reaching agreement is therefore essential for successful collaborations:

Our hospital community geriatric assessment team provides outreach services. We hold a multidisciplinary conference every few months and discuss how to manage cases. Professionals from different disciplines give opinions and suggestions. We also discuss whether we would need to report a case to the authorities if it was someone in a nursing home. (Liz, Medical Social Worker, General Hospital)

I think input from medical doctors and clinical psychologists is important. Their professional assessments can provide relevant information that will help us to confirm whether we have an elder abuse case. A medical doctor would guide the discussion most of the time. If we rule out a case of elder abuse, we work together to see how we can improve patient care. (Liz, Medical Social Worker, General Hospital)

## Effective use of community resources

Intervention in and prevention of elder abuse often involve resource provision and service referrals. Connecting abuse victims with social resources may reduce the risk of further abuse [17]

Besides helping clients adapt and feel psychologically safe at the shelter, we also discuss practical issues. If he/she wants to leave his/her home, we ask integrated family services centers or other social service units to help them to apply to nursing homes or find alternative accommodation. If the client wishes to deal with their family members’ mental health problems, we refer them to mental health services. In addition to individual counseling, we also advise clients to join various activities during their stay with us. (Ofelia, Social Worker, Elder Abuse Shelter)

The hospital does not force older adults to return home after discharge. In cases where grown-up children refuse to take care of older patients, our medical social work team will help provide alternative solutions, private housing or government subsidized nursing homes, or alternative housing. The goal is to minimize the stress experienced by older adults. (Jenny, Nurse, Hospital Accident and Emergency Unit)

### Raising elder abuse awareness

#### Professional training

Awareness of elder abuse among frontline practitioners has been found to be limited [30,17]. Frontline practitioners are medical, social, and healthcare professionals, but also those working in legal domains, the judiciary, and care providers. Ideally, such professionals would have both the necessary knowledge and confidence to identify and intervene in suspected elder abuse cases:

Most frontline staff, such as receptionists, personal care workers, and healthcare workers, provide hands-on care to older persons. They are the first line of workers who are alert to the life circumstances of an older person. Say you visit a home and you can tell from the smell that it has been days since the older person has taken a bath. You can pick up lots of information from the living environment. Training should not be limited to social workers or health professionals; frontline staff, in particular those in supportive roles, should receive training. They spend the most time with clients and are in the best position to identify elder abuse cases. (Ursula, Social Worker, Elderly Services)

Most families eventually call the police. A police taskforce would be an effective way to refer clients to necessary resources if they could identify elder abuse cases. (Gloria, Social Worker, Shelter for Domestic Violence Victims)

Distinguishing the signs of abuse from accidental injuries, physiological changes due to aging, or chronic diseases can be difficult [24,30]. The medical social worker participants suggested that basic medical knowledge can help to differentiate elder abuse from accidental injuries:

Some elder abuse cases take place in nursing homes, among which drug-related incidents represent the majority. Say a resident with no history of diabetes was given diabetic medication and has low blood sugar as a result. It could be an honest mistake, or it could be someone playing a bad joke. At times we also see atypical bone fractures, but the nursing home reports no falls. How likely is it that a bed-bound patient was walking around and ended up falling and breaking a bone? You can’t help but wonder whether someone hit him. I consulted a geriatrician about this. I was told that long-term bed-bound status may result in a patient’s bone being more brittle and easily breakable if too much force is used in transferring a patient. Nurses would call that “rough handling.” As medical social workers, we need more knowledge of bone fractures and injuries. It would make it a lot easier for us to understand the situation, or to collect evidence. (Kate, Medical Social Worker, General Hospital)

Some medical knowledge would come in handy for social workers, in particular medical social workers. A colleague of mine received a case of suspected elder abuse in a nursing home. A family member spotted lots of bruises all over the patient’s body during her visit. The thing is, bruises can result from a host of different conditions. Some basic medical knowledge would be useful in a situation like that. (Xania, Medical Social Worker, General Hospital)

The participants also suggested that involving survivors of elder abuse in professional and community training can be beneficial:

Elder abuse survivors are very keen to share their experience with the police, medical staff, and younger people in the community. In doing so, they not only offer first-hand accounts to trainees but also empower other elder abuse survivors. (Patty, Social Worker, Elder Abuse Shelter)

### Public education

Awareness campaigns can reduce the negative labeling effects of elder abuse and encourage victims to seek help [17]. In some countries, awareness campaigns are regarded as more important than interventions and prevention [7]. Promoting awareness of the rights of the elderly and the definitions, signs and symptoms, and consequences of elder abuse can be aimed at the elderly themselves, caregivers, service professionals, policymakers, stakeholders, and the general public.

Is the older victim aware that he or she is being abused? Not necessarily. This is exactly why we need to deliver more public education. (Wesley, social worker, Elderly Services)

An elder abuse victim may not be aware that he is being abused. Training would need to start by equipping older persons with this basic knowledge, so that they understand their own rights. (Emma, Social Worker, Elderly Services)

To quote domestic violence victims I have worked with: It is easy for physical injuries to heal and recover, much less so for psychological abuse. Being repeatedly told, “You are a useless creature,” “You are better off dead” has a long-term impact on victims in terms of cognition, emotions, etc. We utilize experiential learning in our training programs. For example, we invite participants to sit and have everyone scold him for a couple of minutes, so they can experience the strong feelings resulting from verbal abuse. This exercise is very useful for younger people. They begin to understand how a slip of the tongue can have lasting effects on the recipient. (Gloria, Social Worker, Shelter for Domestic Violence Victims)

### Addressing “by-stander effects” and ambivalence

“Sweep the snow from your own doorstep, don’t worry about the frost on your neighbor’s roof.” This Chinese idiom reflects the entrenched cultural belief that one should not get involved in others’ family affairs. Many therefore refrain from providing advice or offering help to an acquaintance even if elder abuse is suspected [17]. A participant shared this experience:

I ran a community education program years ago. We recruited volunteers to do home visits and educate older persons about elder abuse, ways to communicate with suspected victims, etc. At the briefing session with volunteers, I asked them what they would do if they suspected that an older person was being abused. Some responded that they would ask them to tolerate it! And these volunteers were clients who had themselves used our elder abuse services! This reflects the deep-seated idea that it is wrong to tear families apart. Although it didn’t go as I had imagined, the program gave me a new perspective on how we should mobilize older persons to help their peers, how having a similar upbringing and values probably allows them to communicate better with each other. (Carmen, Social Worker, Elder Abuse Shelter)

## Discussion

As elder abuse can have extremely serious consequences, early detection, identification, and interventions are required. In this focus group study, 32 frontline nursing and social work professionals shared their knowledge and insights concerning elder abuse intervention. They identified the important elements in such interventions and suggested potential ways of improving current initiatives.

First, the participants highlighted the importance of timely detection and identification of abuse. They suggested that screening for abuse in routine services provided to the elderly and in medical settings can help case identification. They noted that it was often difficult to differentiate elder abuse from accidental injuries and suggested involving medical professionals in the screening process. The injury patterns common to abused older adults (Ziminski et al., 2013, [31] have been identified, which can be useful to frontline professionals. Most elder abuse victims choose not to seek help from individuals or formal support services [14,32] due to the influence of traditional Chinese culture, and they may prioritize family honor and harmony over their own safety [17]. Incorporating considerations of abuse in routine assessments can help to identify elder abuse cases.

The participants also noted the importance of engaging stakeholders in elder abuse detection, including health, social service and legal professionals and knowledgeable others in the community, such as neighbors, other family members, and friends. A previous study found that around a quarter of cases are referred by someone from the victim’s social network [17]. Studies outside of Hong Kong also indicate that non-abusing family members, friends, or significant others can encourage victims to seek professional help [14]. The level of organizational support also has a bearing on case identification. Social work in Hong Kong is characterized by inadequate resources and high job stress, caseloads, and turnover rates [33]. Thus, support from organizational management is thus extremely important. The willingness to devote time and effort to identifying elder abuse cases can be encouraged through official recognition that the issue is a priority, policies that support workers investigating such cases, and adequate resource allocation.

In terms of the intervention process, providing tangible support to abuse victims not only meets clients’ needs but has the added value of demonstrating care and support. Tangible support comes in various forms, from financial support to providing shelter, food, and clothing. The participants noted that accepting tangible support also has a symbolic meaning, as the clients are slowly accepting changes in their lives. Active listening and respecting clients’ autonomy and privacy were also identified as essential skills and approaches that help build a trusting, non-threatening relationship with clients that can lead to positive outcomes. The importance of progressing at the clients’ pace and recognizing the extent to which they are ready to change was an interesting point that came out of the discussions. This echoes the emphasis on the voluntary acceptance of intervention, which is critically important for successful outcomes [29]. Some victims suffer for a long time before they are willing to take steps to stop abuse [34], but it is important to consider the clients’ readiness for change and allow them to move at a pace they are comfortable with.

A strength-based approach to elder abuse intervention was emphasized, such as the example of a “buddy program” in which abuse survivors act as peer counsellors. Peer support groups are common and effective in interventions for abuse victims [27]. They can offer empowerment, a sense of safety, social support networks, effective coping methods, personal growth, and companionship [26,27,28]. Previous studies have indicated that the healthy relationships built in support groups are conducive to developing psychological resources and formal help-seeking behavior [14,15,35,17].

The participants also noted that it is important to support caregivers. Other studies have suggested that alleviating the burden experienced by caregivers can in some cases prevent elder abuse [36,7]. Measures including education about the concept of abuse, strategies to control it, and emotional management can be helpful. The broken relationship between victim and perpetrator can be restored if the caregiver is provided with sufficient support [24]. Many families in Hong Kong hire foreign domestic helpers to assist with elderly care, and they often take on day-to-day care such as cooking, cleaning, transferring, bathing, and feeding. Many also experience a language barrier when communicating with care recipients. Providing adequate support to this unique group of caregivers is essential.

Our participants agreed that no single profession should be solely responsible for elder abuse cases, and that multi-disciplinary and cross-agency collaboration is beneficial. Research has indicated that a multidisciplinary approach can enhance the efficiency of service delivery and improve intervention outcomes [25,7]. In addition to traditional human services professionals such as social workers, nurses, medical doctors, and psychologists, participants suggested that involving police officers, legal and judiciary professionals, building managers, housing authorities, and others would be helpful.

Finally, public awareness programs are required to educate senior citizens, caregivers, and families about senior rights and elder abuse, advise potential victims about how and where to seek help, and enable the general public to detect elder abuse. Prevention and intervention thus requires sustained and determined effort.

Our study has several limitations that should be considered when interpreting our findings. First, the participants in the study were referred by organizations that specialize in elder abuse, domestic violence, and emergency medicine. However, those who work in other sectors and may also have experience of working with elder abuse victims, such as occupational therapists, physiotherapists, and medical doctors, are not represented, nor are any other organizations that encounter elder abuse. Further studies should adopt broader inclusion criteria and consider professionals with different backgrounds. Furthermore, while focus groups allow participants to exchange their views and facilitate each other to share their experience in the process, the process could be clouded by social desirability and participants may be reluctant to share personal views that differ from the group norm. Moreover, all focus group in this study consisted of single profession. Further study should consider use of in-depth interviews or focus groups of mixed professions. Finally, our study was conducted online during the COVID-19 pandemic when social distancing measures were in place, rather than through face-to-face focus groups. The levels of participation and interaction may differ in physical meetings.

## Conclusion

To conclude, tailored training is urgently needed to equip frontline professionals with the necessary skills to identify elder abuse cases and to assess the risk of abuse. While clients’ safety and need for tangible support remains a primary concern, frontline professionals should also respect clients’ autonomy and privacy. A client-centered, strength-based approach that involves supportive peers and addresses the complex family relationships involved can be useful. Interventions should also involve cross-discipline and cross-agency collaboration. The responsibility of preventing and intervening in elder abuse should not fall on any single profession or sector, and sustained and collaborative effort from all sectors of society is required to provide a safe environment for senior citizens.

## Data Availability

Data will be available upon request from the corresponding author.
